# Combination therapy design for maximizing sensitivity and minimizing toxicity

**DOI:** 10.1186/s12859-017-1523-1

**Published:** 2017-03-22

**Authors:** Kevin Matlock, Noah Berlow, Charles Keller, Ranadip Pal

**Affiliations:** 1 Department of Electrical and Computer Engineering, Texas Tech University, 1012 Boston Ave, Lubbock, 79409 TX USA; 2 Children’s Cancer Therapy Development Institute, Portland, 97005 OR USA

**Keywords:** Combination drug design, Lexicographic search, Toxicity constraints

## Abstract

**Background:**

Design of personalized targeted therapies involve modeling of patient sensitivity to various drugs and drug combinations. Majority of studies evaluate the sensitivity of tumor cells to targeted drugs without modeling the effect of the drugs on normal cells. In this article, we consider the individual modeling of drug responses to tumor and normal cells and utilize them to design targeted combination therapies that maximize sensitivity over tumor cells and minimize toxicity over normal cells.

**Results:**

The problem is formulated as maximizing sensitivity over tumor cell models while maintaining sensitivity below a threshold over normal cell models. We utilize the constrained structure of tumor proliferation models to design an accelerated lexicographic search algorithm for generating the optimal solution. For comparison purposes, we also designed two suboptimal search algorithms based on evolutionary algorithms and hill-climbing based techniques. Results over synthetic models and models generated from Genomics of Drug Sensitivity in Cancer database shows the ability of the proposed algorithms to arrive at optimal or close to optimal solutions in significantly lower number of steps as compared to exhaustive search. We also present the theoretical analysis of the expected number of comparisons required for the proposed Lexicographic search that compare favorably with the observed number of computations.

**Conclusions:**

The proposed algorithms provide a framework for design of combination therapy that tackles tumor heterogeneity while satisfying toxicity constraints.

## Background

Design of drug therapies for cancer have primarily been considered from the perspective of sensitivity prediction using genetic characterizations as the predictor variables [1–3]. The genetic characterization based methodologies have severe limitations when the cancer type shows numerous aberrations among the samples and consequently predicting sensitivity based on similar steady state genetic characterizations provide limited accuracy. We have recently considered the modeling of tumor sensitivity using functional drug response data [4, 5], along with a functional and genetic characterization based integrated modeling [6]. Models were designed based on the in vitro tumor response to a set of drugs with known targets. However, the combination therapy design was based on the model reflecting the average behavior of the tumor tissue [7].

In this article, we incorporate heterogeneity by considering the effect of a drug on various parts of the tumors and incorporate toxicity by considering the effect of the drug on normal cell types. Consider a solid tumor tissue where the biopsy sample can be divided into separate samples to explore the heterogeneity. We can pass each biopsy sample through a drug screen to create a probabilistic target inhibition map (PTIM) [5] model. Let *M* *T*_1_,*M* *T*_2_,⋯,*M* *T*_*k*_ denote the *k* models corresponding to the *k* spatial tumor biopsies.

For toxicity the affect of the drug is not limited to the same organ that the tumor resides in. To solve this we can pass normal cell cultures from different organs of the body through drug screens to create separate models of different organs to assess toxicity of the drugs. Note that, normal cells from kidney, lungs etc. of a specific cancer patient may not be readily available and thus, the response to drugs can be approximated by using drug screens on normal human based cell lines of kidney, lungs and other organs. The assumption is that variations in normal cell response to different drugs over different patients are smaller compared to tumor cell response over different patients. The normal cell response to different drugs can vary significantly for cells belonging to different organs in the body. Let *M* *N*_1_,*M* *N*_2_,⋯,*M* *N*_*p*_ denote *p* models corresponding to *p* different normal cell types.

The goal of the combination therapy design will be to select a set of drugs that will maximize the sensitivity over heterogeneous tumor models *M* *T*_1_,*M* *T*_2_,⋯,*M* *T*_*k*_ and minimize sensitivity over normal cell type models *M* *N*_1_,*M* *N*_2_,⋯,*M* *N*_*p*_. Note that currently available combination therapy design techniques are model free and require multiple experimental iterations to arrive at the optimal strategy [8–15]. This article considers model based combination therapy design over multiple models of tumor and normal cell lines.

We utilize the constrained structure of tumor proliferation models to design an accelerated lexicographic search algorithm for generating the optimal solution. For comparison purposes, we also designed two suboptimal search algorithms based on evolutionary algorithms and hill-climbing based techniques. We test the performance of our algorithms on synthetic models and models generated from Genomics of Drug Sensitivity in Cancer (GDSC) database [16]. Utilizing the model structure in the search process allows us to arrive at optimal or close to optimal solutions in significantly lower number of steps as compared to exhaustive search. The article also presents the theoretical analysis of the expected number of comparisons required for the proposed optimal Lexicographic search that compare favorably with the observed number of computations.

The paper is organized as follows: The model representation is discussed in “Methods” section. “Algorithms” section discusses the proposed lexicographic search algorithm along with suboptimal Genetic algorithm and Hill climbing approaches. “Results and discussion” section presents the results followed by Conclusions in “Conclusions” section.

## Methods

### Model type

**Fig. 1:**
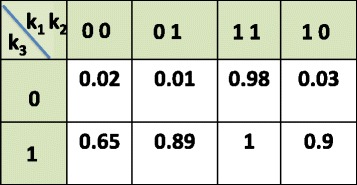
PTIM example. An example PTIM model with 3 targets *k*_1_, *k*_2_ and *k*_3_

**Fig. 2:**
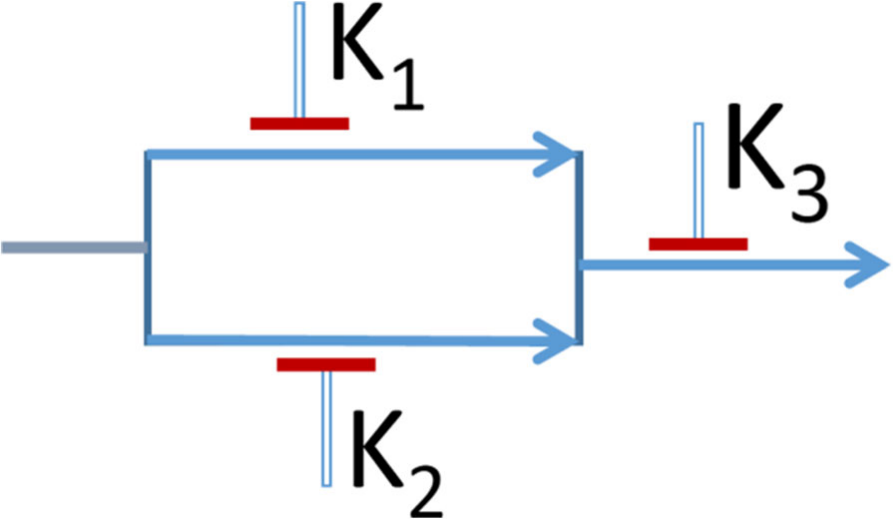
PTIM circuit. A circuit representation of a PTIM model in Fig. 1

In this section, we provide a brief review of the model used to represent each spatial tumor biopsy or normal cell line. A Probabilistic Target Inhibition Map (PTIM) model provides an estimate of sensitivity for all possible target inhibitions. Consider the example PTIM model with 3 targets *k*_1_, *k*_2_, *k*_3_ shown in Fig. 1 where the values for each cell represent the sensitivity corresponding to that specific inhibition. For instance, inhibition of *k*_3_ alone will produce a sensitivity of 0.75. Since the commonly used targeted drugs inhibit oncogenes, we consider the targets to be all oncogenes and inhibition of more oncogenes can only cause the sensitivity to remain same or increase. For instance, since the inhibition of *k*_3_ alone produces a sensitivity of 0.75, all supersets of that inhibition ([*k*_3_,*k*_1_], [*k*_3_,*k*_2_], [*k*_3_,*k*_1_,*k*_2_]) will have sensitivity ≥0.75. Similarly, any subset of known inhibition will have sensitivity less than or equal to the observed value. Based on these two biological constraints and limited drug perturbation experiments, we can arrive at an inferred PTIM model that can provide an estimate of sensitivity for all possible target inhibitions. The details of the model are available at [4–6] along with biological validation at [17]. Note that a PTIM can also be approximately represented as a tumor proliferation circuit as shown in Fig. 2 where the tumor proliferation can be restricted by inhibiting at least one series block. For instance, inhibition of the [*k*_1_,*k*_2_] block will provide a sensitivity of 0.95 whereas inhibition of *k*_3_ will provide a sensitivity of 0.75. Inhibiting more than the minimum will produce higher sensitivities that are given by the original map shown in Fig. 1.

### Structure of tumor and normal cell models

**Fig. 3:**
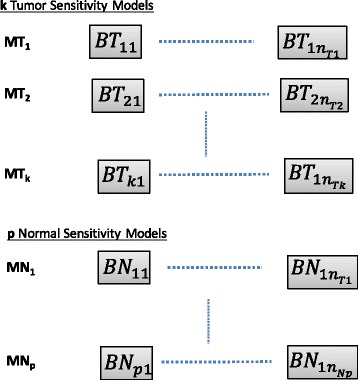
PTIM block diagram. Representation of *k* tumor models and *p* normal models as series of parallel target blocks

Based on the previously discussed model structure, each of the *k* tumor models will be represented as a probabilistic target inhibition map that can also be approximated by a circuit representation of series of parallel blocks as shown in Fig. 3.

In Fig. 3, the number of blocks for models *M* *T*_*i*_ for *i*=1,⋯,*k* and *M* *N*_*j*_ for *j*=1,⋯,*p* are denoted by *n*_*Ti*_ and *n*_*Nj*_ respectively. Every model is composed of five blocks connected in series. Each block, *b*, contains a set of targets *T*_*bi*_ (up to a maximum of 5 targets), that are connected in parallel. Thus each model can have up to 25 targets.

### Optimization objectives

For optimization, we consider both the worst case and best expected scenario. Let *O*(*M* *T*_*i*_,*ϕ*) denote the sensitivity of Tumor model *i* for *i*=1,⋯,*k* with inhibition *ϕ*. Let *O*(*M* *N*_*i*_,*ϕ*) denote the sensitivity of normal model *i* for *i*=1,⋯,*p* with inhibition *ϕ*.

**Worst case optimization (WCO):** We desire high sensitivity over the tumor cell lines and low sensitivity over the normal cells which can be formulated in the worst case scenario as maximizing the minimum sensitivity over the tumorous cells while maintaining the maximum sensitivity over the normal cells below a certain threshold *θ*_1_.

i.e. max*ϕ*(min*i*[*O*(*M* *T*_*i*_,*ϕ*)]) while max*i* [ *O*(*M* *N*_*i*_,*ϕ*) ] ≤*θ*_1_

**Best expected optimization (BEO):** In this scenario, our goal will be to maximize the average sensitivity over the tumorous cells while maintaining the average sensitivity over the normal cells below a threshold *θ*_2_ i.e. $ \max _{\phi } (\overline {O({MT}_{i}, \phi)})$ while $\overline {O({MN}_{i}, \phi)} \leq \theta _{2}$.

## Algorithms

### Lexicographic search algorithm

In order to find the optimal target set for our problem, we can exhaustively search through all possible target combinations for a given toxicity threshold. Normally, for *T* targets this would require searching through 2^*T*^ combinations, which is not computationally feasible for large *T*. However, we can utilize the monotonic relationships of PTIM models to our advantage to reduce the number of search steps. Given a set of targets *S*_1_ whose toxicity (i.e. sensitivity over normal cell lines) is greater than a given threshold *θ*_1_, then all possible supersets of *S*_1_ will also have a toxicity ≥*θ*_1_ and thus, there is no need to search through the supersets of *S*_1_. Note that this is only valid when all the targets are oncogenes.



**Fig. 4:**
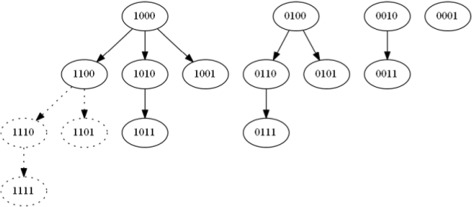
Lexicographic search example. Lexicographical Search for 4 Targets. Utilizing the superset rule, sets surrounded by *dotted lines* are excluded from the search process when toxicity of [1100] ≥*θ*_1_

To take advantage of this property, we perform a branching Lexicographical Search of the solution space. We can view the solution space as a directed graph where each node of the graph is our target set represented as a binary string with *T* bits. Each edge of the graph corresponds to turning on one bit to the right of the least significant bit, creating a superset of that node. If the toxicity at a node exceeds the threshold, then there is no need to continue along the associated edges and we should instead trace back to the previous node. A recursive algorithm to perform this search is shown in Algorithm 1. A demo using four targets is shown in Fig. 4. Note that in Fig. 4, we are assuming that the sensitivity over normal cell lines exceed the given threshold for target set [1100] and thus its supersets consisting of [1110], [1101] and [1111] marked by dotted lines are excluded from the search process.

### Lexicographic search analysis

In this section, we consider the stochastic analysis of the proposed Lexicographic Search to generate the expected number of searches. Let *C* *D* *F*(*l*,*θ*)=*f*(*l*,*θ*) denote the probability that a normal cell model will have sensitivity ≤*θ* for a random inhibition of *l* targets. We then define *g*_*P*_(*l*,*θ*) as the probability that we will not exceed sensitivity *θ* while targeting *l* random inhibitions in a set of *P* cells. For the worst case scenario, $g^{wco}_{P}(l,\theta)$ is the probability that the maximum sensitivity over the normal cells is below threshold *θ*. Considering, independence of the normal cell sensitivities, we have:

1 $$\begin{array}{*{20}l} g^{wco}_{P}(l,\theta) &=Pr({max}_{i}(O({MT}_{i})) \leq \theta)  \\ &= Pr(O({MT}_{1}) \leq \theta, O({MT}_{2}) \leq \theta, \cdots, \\ &\quad\; O({MT}_{P}) \leq \theta)  \\ &= Pr(O({MT}_{1}) \leq \theta) \bullet Pr(O({MT}_{2}) \leq \theta) \cdots  \\ &\;\;\;\; \bullet Pr(O({MT}_{P}) \leq \theta)  \\ &= f(l,\theta)^{P} \end{array} $$

For the best expected scenario, let us consider the probability density function of observing a sensitivity of *θ* after *l* inhibitions $PDF(l,\theta) = \frac {\partial f(l,\theta)}{\partial \theta }$. Let *X* denote the random variable with *P* *D* *F*(*l*,*θ*) and *Z* denote the sum of *P* such random variables. The probability density function of *Z* denoted by *q*_*P*_(*l*,*θ*) can be calculated by repeatedly convolving *P* *D* *F*(*l*,*θ*) with itself for *P*−1 times and is given by

$$ q_{P}(l,\theta) = PDF(l,\theta) \ast PDF(l,\theta) \dots \ast PDF(l, \theta) $$

Let *Y* denote the random variable denoting the average sensitivity over *P* cell lines with *l* random inhibitions. The probability density function of *Y* is given by

2 $$\begin{array}{*{20}l} {pdf}_{Y}(\theta) &= h_{p}(l,\theta) = P * q_{P}(l, P\theta) \end{array} $$

We can then estimate the cumulative density function of *Y*, $g^{beo}_{P}(l,\theta)$ by integrating across *θ*:

$$ g^{beo}_{P}(l,\theta) = \int_{0}^{\theta} h_{P}(l,u) du $$

#### Expected savings

Define *A*_*i*_ to denote the event that the sensitivity over *P* normal models with *i* random inhibitions ≥*θ*, i.e. *P* *r*(*A*_*i*_)=1−*g*_*P*_(*i*,*θ*). Let *L*_*i*_ denote the event of stoping at level *i* of the Lexicographic Search where *i* represents the number of bits we are searching through. The probability of event *L*_*i*_ is given by:

3 $$\begin{array}{*{20}l} P(L_{i}) & = P\left(A_{i} \cap A_{i-1}^{C} \cap A_{i-2}^{C} \dots \cap A_{1}^{C}\right)  \\ & = P\left(A_{i} | \bigcap_{j=1}^{i-1} A_{j}^{C}\right) P\left(\bigcap_{j=1}^{i-1} A_{j}^{C}\right)  \end{array} $$

We note that:

4 $$ P\left(A_{i} | \bigcap_{j=1}^{i-1} A_{j}^{C}\right) = 1 - P\left(A_{i}^{C} | \bigcap_{j=1}^{i-1} A_{j}^{C}\right)   $$

By applying Bayes’ theorem, we can simplify further:

5 $$\begin{array}{*{20}l}{} P\left(A_{i}^{C} | A_{i-1}^{C} \cap A_{i-2}^{C} \cap \dots A_{1}^{C}\right) &= \frac{P\left(A_{i}^{C}\right)P\left(\bigcap_{j=1}^{i-1} A_{j}^{C}|A_{i}^{C}\right)}{P\left(\bigcap_{j=1}^{i-1} A_{j}^{C}\right)}  \\ &= \frac{P\left(A_{i}^{C}\right)}{P\left(\bigcap_{j=1}^{i-1} A_{j}^{C}\right)}  \end{array} $$

By combining Eqs. 3, 4 and 5, we have:

6 $$ P(L_{i}) = g(i-1,\theta) - g(i,\theta)  $$

To find the expected savings, we note that by stopping at *L*_*i*_, we search through $\sum _{j=0}^{i} {{T}\choose {j}}$ combinations. Thus, the expected savings *E*(*S*) is given by :

7 $$\begin{array}{*{20}l} E(S) &= \sum_{i=1}^{T}P\left(A_{i} \cap A_{i-1}^{C} \cap \dots A_{1}^{C}\right)\left[2^{T} - \sum_{j=0}^{i} {{T}\choose{j}}\right] \end{array} $$

8 $$\begin{array}{*{20}l} &= \sum_{i=1}^{T}\left[g(i-1,\theta) - g(i,\theta)\right]\left[2^{T} - \sum_{j=0}^{i} {{T}\choose{j}}\right] \end{array} $$

### Genetic algorithm based search

#### Pareto optimality

We consider a multi-objective optimization scenario where we maximize sensitivity over tumor cells and minimize sensitivity over normal cells. For worst case optimization scenario, if therapies *ϕ*_1_ and *ϕ*_2_ satisfy the following relation: *m* *i* *n*_1≤*i*≤*k*_[ *O*(*M* *T*_*i*_,*ϕ*_1_)]≥*m* *i* *n*_1≤*i*≤*k*_[*O*(*M* *T*_*i*_,*ϕ*_2_)] and *m* *a* *x*_1≤*i*≤*p*_[ *O*(*M* *N*_*i*_,*ϕ*_1_)]≤*m* *a* *x*_1≤*i*≤*p*_[ *O*(*M* *N*_*i*_,*ϕ*_2_)] with either *m* *i* *n*_1≤*i*≤*k*_[*O*(*M* *T*_*i*_, *ϕ*_1_)]>*m* *i* *n*_1≤*i*≤*k*_[*O*(*M* *T*_*i*_,*ϕ*_2_)] or *m* *a* *x*_1≤*i*≤*p*_[*O*(*M* *N*_*i*_,*ϕ*_1_)]<*m* *a* *x*_1≤*i*≤*p*_[*O*(*M* *N*_*i*_,*ϕ*_2_)], then therapy *ϕ*_1_ is considered to dominate *ϕ*_2_ from the multi-objective Pareto sense. The therapies that are not dominated by any other therapy will form the Pareto efficient front.

#### Algorithm

Genetic Algorithms (GA) are inspired by evolutionary theory where strong species have a higher opportunity to pass their genes to offspring via reproduction and weaker chromosomes are eliminated by natural selection [18, 19]. Each generation or population consists of diverse individuals or chromosomes and in our Genetic Algorithm based Combination Therapy design (GACT), each therapy *ϕ* is regarded as a chromosome comprised of different target inhibitions. These target inhibitions are binary variables with values of 0 (non-inhibited) or 1 (completely inhibited). In order to select the best solutions (therapies) for the next generation, the fitness of each solution is computed. The therapies with the best fitness (our Pareto front) will be selected as the parents of the next generation. During each reproduction process, crossover and mutation operators are applied for the purpose of generating new solutions from existing ones. Mutation is performed by randomly flipping inhibition values of our targets. Crossover is performed by randomly picking values between two different target sets. For example, if we take the two target sets [*a*_1_,*a*_2_,*a*_3_] and [*b*_1_,*b*_2_,*b*_3_] a crossover between them can be performed by considering:

$$[c_{1}, c_{2}, c_{3}] = [a_{1}, b_{2}, a_{3}] $$

**Fig. 5:**
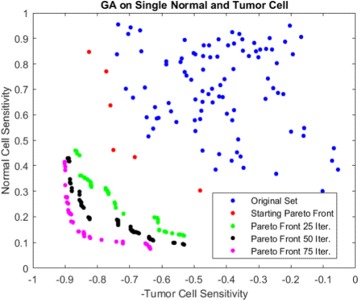
GACT example. Pareto fronts converge for subsequent genetic algorithm iterations



Based on our starting set of targets (*M*), we form the initial population *P*_0_ of *N* random target inhibition profiles. After calculating the fitness functions for the existing population, we calculate different Pareto front layers according to their dominance relationships. The top Pareto optimal points are selected to pairwise conduct crossover and mutations to form offsprings. Here we have set the number of offspring to be at least twice the number of points in our Pareto fronts with a minimum of *O* *f* *f* *m* *i* *n*=1000 and a maximum of *n* *O* *f* *f* *m* *a* *x*=15,000 offsprings. After merging these offsprings with their parent population *P*_*t*−1_, we extract top *N* therapies to generate population *P*_*t*_. We iterate our algorithm until we have achieved *totalG* generations or the number of offspring is greater than *nOffmax*. Note that evolutionary algorithms like GA will not guarantee convergence of the Pareto front but the performance of our therapies will improve if the Pareto front moves towards our desired direction with subsequent GA iterations. The detailed procedure for multi-objective GACT is shown as Algorithm 2. Figure 5 illustrates how the algorithm moves our pareto front towards better solutions across successive iterations. After running the GACT, we consider the final Pareto front and pick the target set that provides the maximum sensitivity over tumor cell lines when the toxicity is below threshold *θ*_1_.

### Random restart hill climbing

We consider an additional suboptimal algorithm based on Hill Climbing to search the target space. Hill Climbing is an iterative method for finding the local maximum for any arbitrary function. Given a starting point, the algorithm considers all the nearest neighbors and then selects the neighbor that provides the best solution for the given optimization criteria. These steps are subsequently repeated until there are no neighbors that provide a better solution or a maximum number of iterations have been reached. While simple and effective in finding local optimum, Hill Climbing will rarely find the global optimum for non-convex functions. In order to overcome this handicap, we will randomly restart our search to a new random position whenever the algorithm converges to a local optimum.

In our case, the starting criteria will be a random set of targets chosen using latin hypercube numbers and each neighbor will be created by inhibiting or un-inhibiting a single target. As shown in Algorithm 3, our optimization criteria will change depending on the toxicity of our current set. If the toxicity is greater than the given threshold then we choose the neighbor with the least toxicity, otherwise we pick the neighbor with the highest sensitivity whose toxicity is below the threshold. When no improvements can be found among the neighbors we randomly choose a new set of targets. This is repeated until we have completed *maxIter* iterations.



## Results and discussion

To evaluate the performance of our algorithms, we considered both synthetic models and models based on experimental datasets.

### Synthetic model generation

The synthetic models are simulated using a proliferation network structure based on probabilistic target inhibition maps [5, 7]. Each cellular pathway, *i*, representing either a tumor or normal cell model is modelled by connecting a set of blocks in series. The number of blocks for models *M* *T*_*i*_ for *i*=1,⋯,*k* and *M* *N*_*j*_ for *j*=1,⋯,*p* are denoted by *n*_*Ti*_ and *n*_*Nj*_ respectively. Within each block, *b*, contains a set of targets *T*_*bi*_ (up to a maximum of 5 targets), that are connected in parallel. Since the targets are in parallel, the effective inhibition for each block given a set of target inhibitions, *ϕ*, is the minimum inhibition of the given targets within the block. Thus, the effective inhibition of block *b* in model *M* *T*_*i*_ with target inhibition *ϕ* is given by *λ*(*M* *T*_*i*_,*b*,*ϕ*)= min(∀*ϕ*∈*T*_*bi*_).

Each block is also given a score, *S*_*bi*_, randomly using a uniform distribution with a minimum of 0.5 and maximum of 1. Finally, the overall sensitivity of the pathway can be computed using the following equation where we assume independence between the series blocks:

$$Sensitivity({MT}_{i},\phi) = 1-\prod_{b=1}^{N_{Ti}}(1-S_{bi} \lambda ({MT}_{i},b,\phi)) $$

Similary for normal cells:

$$Sensitivity({MN}_{i},\phi) = 1-\prod_{b=1}^{N_{Ni}}(1-S_{bi} \lambda ({MN}_{i},b,\phi)) $$

A representation of *k* tumor and *p* normal models as series of parallel target blocks is shown in Fig 3.

The synthetic model set consists of a total of 1000 synthetic pathways, 500 normal and 500 cancerous. A total of 25 targets are examined and all targets are equiprobable in both the cancer and normal pathways. We group the pathways into 100 groups where each group has 5 normal pathways and 5 cancerous pathways. From every group, we consider *nNormal* normal pathways and *nTumor* cancerous pathways.

### GDSC data

In order to test our algorithms on biological functional data, we have utilized the GDSC database [16] to generate a set of PTIM models for 20 different cell lines. These cell lines were segregated into groups of 10, the first group is composed of breast-cancer cell lines and the second group is B-cell lymphoma cancer cell lines. A list of the cell lines is shown in Table 1. For each of the cell lines, we considered the IC50 values for 32 drugs and combined with the corresponding drug panels generated a PTIM model. The drug panels contained the *K*_*d*_ values for 404 targets and 62 of these targets were found to correspond to the PTIM model of at least one of the cell lines, 42 targets in the breast cell lines, 49 in the lymphoma and 27 targets where found in both the breast and lymphoma cell lines.

**Table 1:** Cell lines used in GDSC dataset

Breast	Lymphoma
BT474	A3KAW
COLO824	A4FUK
DU4475	BC1
EVSAT	CTB1
HCC1187	DOHH2
HCC2157	HT
HCC2218	KARPAS422
MFM223	MC116
MRKnu1	RL
OCUBM	TUR

For the generation of sets of tumor and normal models, we generate 100 groups and for each group we randomly assign one type of cell lines (breast or lymphoma) to be the *Normal* cells and the other type to be the *Tumor* cells. We then randomly pick *nNormal* cell lines from the corresponding group and assign them as *Normal* cell models. Likewise, we pick *nTumor* random cell lines from the other group and assign them as our *Tumor* cell models.

### Performance comparisons

For both the synthetic and GDSC cases, we select the solution that provides the maximum cancer cell sensitivity while keeping the normal cell sensitivity below a threshold of *θ*=0.1 using both the best expected scenario and the worst case optimization method. The maximum cancer sensitivity is then averaged across all 100 groups.

#### GACT parameter selection

In order to find an optimal *r* *a* *t* *e*_*m*_ and *r* *a* *t* *e*_*c*_ for the GACT, we set *n* *T* *u* *m* *o* *r*,*n* *N* *o* *r* *m* *a* *l*=5 and *t* *o* *t* *a* *l* *G*=100. We then repeated the WCO GACT on the synthetic dataset, varying either *r* *a* *t* *e*_*c*_ or *r* *a* *t* *e*_*m*_ on each run. The results are shown in Table 2. Based on these results, we have selected a *r* *a* *t* *e*_*m*_ of 0.2 and *r* *a* *t* *e*_*c*_ of 0.6. We then start varying *nGen* and the corresponding results are recorded in Table 3. From these results we can see that past 400 iterations, there is no significant improvement in the GACT.

**Table 2:** Worst case optimization tumor cell sensitivity using GACT for sythetic models

		*r* *a* *t* *e*_*c*_			
		0.6	0.7	0.8	0.9
*r* *a* *t* *e*_*m*_	0.02	0.5826	0.5891	0.5933	0.5643
	0.05	0.5903	0.5842	0.5877	0.5753
	0.1	0.5770	0.5829	0.5757	0.5728
	0.2	0.5938	0.5923	0.5848	0.5812
	0.5	0.5669	0.5673	0.5762	0.5796

**Table 3:** WCO GACT for synthetic models with varying nGen

*nGen*	*Sensitivity*
100	0.5938
200	0.6009
300	0.6027
400	0.6094
500	0.6074

#### Hill climbing parameter selection

We perform a similar operation on the Hill Climbing algorithm where we keep *n* *T* *u* *m* *o* *r*=*n* *N* *o* *r* *m* *a* *l*=5 and vary *nIter* and measuring the best expected outcome in contrast to worst case optimization in previous case. The results are shown in Table 4. Since, there are no significant changes in performance after 15,000 iterations, we use *m* *a* *x* *I* *t* *e* *r*=15000 for running our Hill Climbing algorithm.

**Table 4:** Hill climbing BEO performance with varying number of iterations

*maxIter*	*Sensitivity*
10000	0.8029
15000	0.8136
20000	0.8214
25000	0.8248
30000	0.8275

### WCO results

The results for worst case optimization for the three approaches on synthetic and biological models are shown in Tables 5, 6, 7, 8, 9 and 10. For the synthetic dataset, we achieved minimum cancer sensitivities >0.60 using the LS (Table 5) and GACT (Table 6) algorithms while the Hill Climbing algorithm could only achieve a sensitivity of 0.55 (Table 7). Since the LS algorithm is the optimal approach, it produces the best performance followed closely by the GACT algorithm for synthetic dataset. However, for the GDSC dataset, the GACT was only able to achieve a sensitivity of 0.33 which is significantly worse than the LS which was able to achieve a sensitivity of 0.45. For the GDSC dataset, it appears that GACT is close to the optimal solution for smaller number of normal cells and increasing the number of normal cells reduces its performance (Table 9). Hill Climbing has reasonable performance for GDSC data based models (Table 10).

**Table 5:** LS worst case optimization synthetic data

		*nNormal*				
		1	2	3	4	5
*nTumor*	1	0.9934	0.9828	0.9633	0.9681	0.9096
	2	0.9867	0.9603	0.9099	0.9255	0.7852
	3	0.9794	0.9454	0.8836	0.8110	0.7331
	4	0.9681	0.9255	0.8487	0.7728	0.6858
	5	0.9607	0.9005	0.8113	0.7194	0.6157

**Table 6:** GACT worst case optimization synthetic data

		*nNormal*				
		1	2	3	4	5
*nTumor*	1	0.9934	0.9828	0.9633	0.9384	0.9096
	2	0.9862	0.9599	0.9085	0.8594	0.7843
	3	0.9772	0.9439	0.8811	0.8007	0.7268
	4	0.9647	0.9200	0.8430	0.7679	0.6717
	5	0.9561	0.8938	0.8056	0.7007	0.6097

**Table 7:** Hill climbing worst case optimization synthetic data

		*nNormal*				
		1	2	3	4	5
*nTumor*	1	0.9926	0.9817	0.9614	0.9369	0.9086
	2	0.9805	0.9550	0.9031	0.8449	0.7635
	3	0.9625	0.9270	0.8650	0.7929	0.7016
	4	0.9402	0.8923	0.8089	0.7341	0.6241
	5	0.9137	0.8514	0.7630	0.6573	0.5536

**Table 8:** LS worst case optimization GDSC data

		*nNormal*				
		1	2	3	4	5
*nTumor*	1	0.9792	0.9536	0.9218	0.8505	0.8282
	2	0.9601	0.9226	0.8718	0.7853	0.7556
	3	0.9326	0.8767	0.8207	0.7017	0.6270
	4	0.9163	0.8280	0.7405	0.6126	0.4880
	5	0.9045	0.7978	0.6809	0.5535	0.4539

**Table 9:** GACT worst case optimization GDSC data

		*nNormal*				
		1	2	3	4	5
*nTumor*	1	0.9789	0.9536	0.8738	0.7532	0.6056
	2	0.9598	0.9124	0.8121	0.6900	0.4951
	3	0.9320	0.8688	0.8062	0.6401	0.4843
	4	0.9152	0.8210	0.7130	0.5525	0.3458
	5	0.9028	0.7905	0.6551	0.4735	0.3348

**Table 10:** Hill climbing worst case optimization GDSC data

		*nNormal*				
		1	2	3	4	5
*nTumor*	1	0.9788	0.9390	0.8859	0.8489	0.8230
	2	0.9588	0.8762	0.8163	0.7399	0.7074
	3	0.9357	0.8283	0.7283	0.6215	0.5724
	4	0.9148	0.7773	0.6533	0.5286	0.4683
	5	0.8959	0.7422	0.5936	0.4584	0.3908

### BEO results

The results for best expected optimization scenario for the three algorithms on synthetic and biological models are shown in Tables 11, 12, 13, 14, 15 and 16. For the synthetic dataset, we achieved expected sensitivities over cancer models higher than 0.85 using the LS (Table 11) and GACT (Table 12) algorithms and >0.81 for Hill Climbing Approach (Table 13). For the GDSC based models, performance close to LS (Table 14) is observed with GACT (Table 15) followed by Hill Climbing (Table 16).

**Table 11:** LS best expected optimization synthetic data

		*nNormal*				
		1	2	3	4	5
*nTumor*	1	0.9934	0.9828	0.9633	0.9384	0.9096
	2	0.9916	0.9756	0.9468	0.9170	0.8744
	3	0.9901	0.9734	0.9461	0.9110	0.8698
	4	0.9883	0.9702	0.9355	0.9049	0.8662
	5	0.9872	0.9668	0.9305	0.8984	0.8596

**Table 12:** GACT best expected optimization synthetic data

		*nNormal*				
		1	2	3	4	5
*nTumor*	1	0.9934	0.9828	0.9632	0.9384	0.9095
	2	0.9912	0.9755	0.9451	0.9156	0.8741
	3	0.9889	0.9716	0.9443	0.9102	0.8664
	4	0.9861	0.9679	0.9325	0.9024	0.8640
	5	0.9830	0.9629	0.9270	0.8945	0.8549

**Table 13:** Hill climbing best expected optimization synthetic data

		*nNormal*				
		1	2	3	4	5
*nTumor*	1	0.9923	0.9817	0.9621	0.9377	0.9069
	2	0.9853	0.9708	0.9409	0.9085	0.8524
	3	0.9779	0.9597	0.9305	0.8900	0.8496
	4	0.9684	0.9485	0.9138	0.8783	0.8268
	5	0.9603	0.9361	0.9016	0.8628	0.8123

**Table 14:** LS best expected outcome GDSC data

		*nNormal*				
		1	2	3	4	5
*nTumor*	1	0.9792	0.9585	0.9315	0.8681	0.8572
	2	0.9783	0.9615	0.9360	0.8909	0.8813
	3	0.9737	0.9568	0.9366	0.8910	0.8653
	4	0.9735	0.9511	0.9263	0.8851	0.8508
	5	0.9736	0.9498	0.9219	0.8826	0.8544

**Table 15:** GACT best expected optimization GDSC data

		*nNormal*				
		1	2	3	4	5
*nTumor*	1	0.9789	0.9584	0.8916	0.8474	0.8129
	2	0.9781	0.9611	0.9357	0.8609	0.8672
	3	0.9734	0.9478	0.8996	0.8709	0.8246
	4	0.9732	0.9502	0.8975	0.8771	0.8328
	5	0.9733	0.9402	0.9124	0.8733	0.8407

**Table 16:** Hill climbing best expected optimization GDSC data

		*nNormal*				
		1	2	3	4	5
*nTumor*	1	0.9777	0.9447	0.9030	0.8600	0.8464
	2	0.9738	0.9360	0.8995	0.8589	0.8286
	3	0.9734	0.9369	0.8956	0.8543	0.8267
	4	0.9731	0.9370	0.8956	0.8501	0.8152
	5	0.9731	0.9380	0.8934	0.8502	0.8177

### Computational complexity

Tables 17 and 18 shows the average number of searches and runtime for each algorithm for synthetic and GDSC datasets respectively. All results are for *n* *N* *o* *r* *m* *a* *l*=*n* *T* *u* *m* *o* *r*=5. Each program is written using MATLAB and ran on a Inspiron 15 laptop with a Core i5-6300HQ processor with 8 GB of RAM. For the synthetic models, GACT was the fastest, taking less than a second for both WCO and BEO. The second fastest was the Hill Climbing (HC) algorithm that takes around 20 s per group while the LS algorithm takes the longest at over 125 s per group. It should be noted that the GACT is able to perform a proportionally larger number of searches in a shorter amount of time than both the LS and HC algorithm. This is because the GACT uses a small number of iterations but performs a large number of searches per iteration and is thus able to be vectorized more than the other algorithms.

**Table 17:** Computational complexity synthetic data

	WCO			BEO		
	Searches	Avg.	Searches	Searches	Avg.	Searches
		Time (s)	per s		Time (s)	per s
LS	1033500	132.2	7814	1033500	126.3	7934
GACT	501000	0.6052	827825	501000	0.5806	862900
HC	380130	20.11	18902	378940	16.69	22705

**Table 18:** Computational complexity GDSC data

	WCO			BEO		
	Searches	Avg.	Searches	Searches	Avg.	Searches
		Time (s)	per s		Time (s)	per s
LS	879	0.095	9253	2032	0.1317	15429
GACT	146430	4.32	33896	30850	1.01	30433
HC	300650	13.82	21749	299580	11.41	26256

In contrast for the GDSC dataset, the LS algorithm performs the fastest due to the small number of searches required. The GACT is now the second fastest and HC is the slowest. It should be noted that the GACT performs significantly slower in the GDSC dataset than the synthetic case. This is because we are unaware of the underlying circuit models of the PTIMs in the GDSC dataset as the sensitivities are computed using a lookup table and cannot be vectorized as efficiently as the synthetic data. In the next section, we explain the smaller number of searchers required for the LS approach for GDSC data as compared to Synthetic data based on the the specific structures of *g*(*l*,*θ*).

### Estimated number of searches

**Fig. 6:**
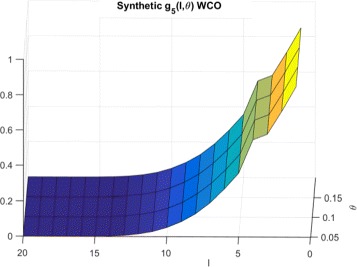
CDFs for synthetic cells and WCO

**Fig. 7:**
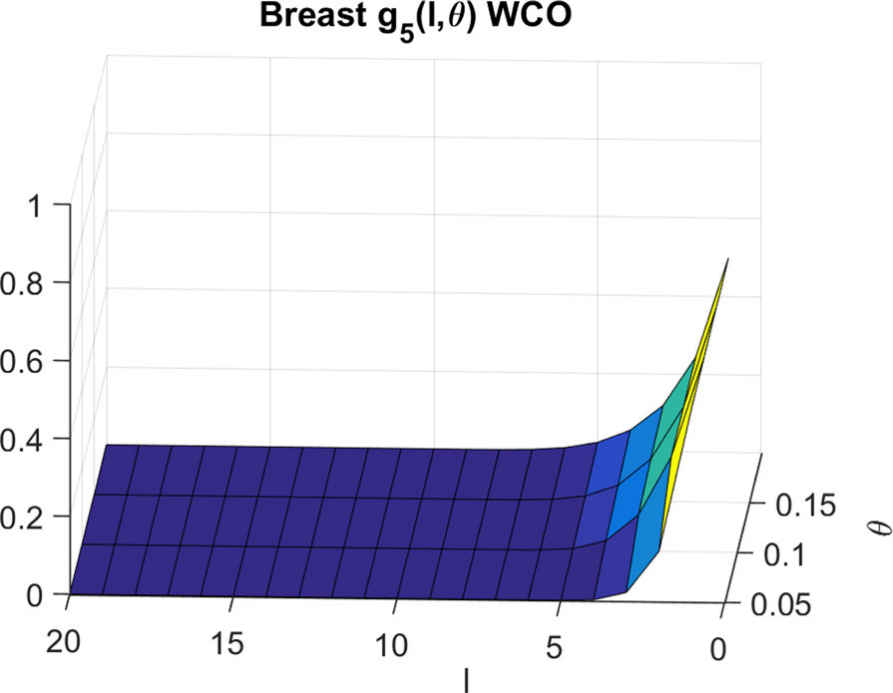
CDFs for breast cells and WCO

**Fig. 8:**
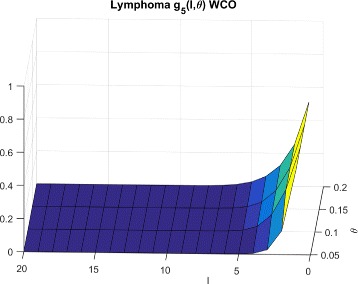
CDFs for lymphoma cells and WCO

In this section, we empirically generate the distributions of searches required for LS technique for random sets of normal and cancerous cells for both synthetic and GDSC data and compare them to theoretical estimates. In order to generate *f*(*l*,*θ*), we randomly select *l* targets and record the measured sensitivity for each cell line following inhibition of the selected *l* targets. This process is repeated 300,000 times for *l*=1 to *nTargets* to generate the entire CDF. The $g^{wco}_{P}(l,\theta)$ distributions for Synthetic, Breast cancer and Lymphoma cell lines are shown in Figs. 6, 7 and 8 respectively.

**Fig. 9:**
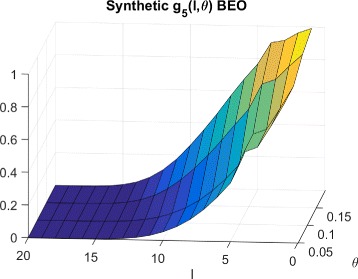
CDFs for synthetic cells and BEO

**Fig. 10:**
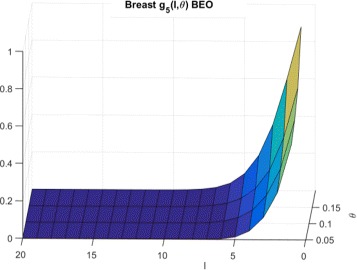
CDFs for breast cells and BEO

**Fig. 11:**
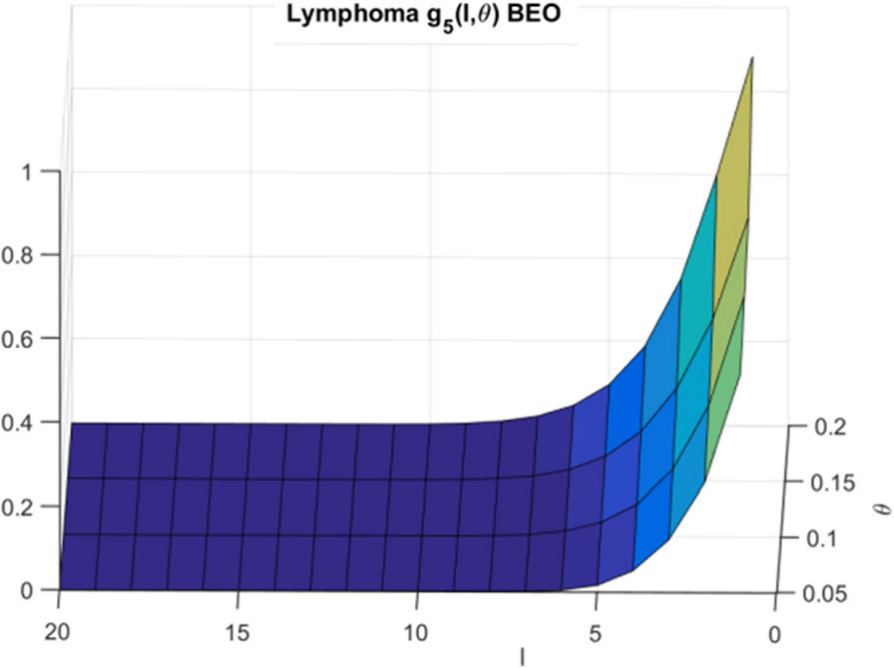
CDFs for lymphoma cells and BEO

The corresponding $g^{beo}_{P}(l,\theta)$ distributions for Synthetic, Breast cancer and Lymphoma cell lines are shown in Figs. 9, 10 and 11 respectively.

**Fig. 12:**
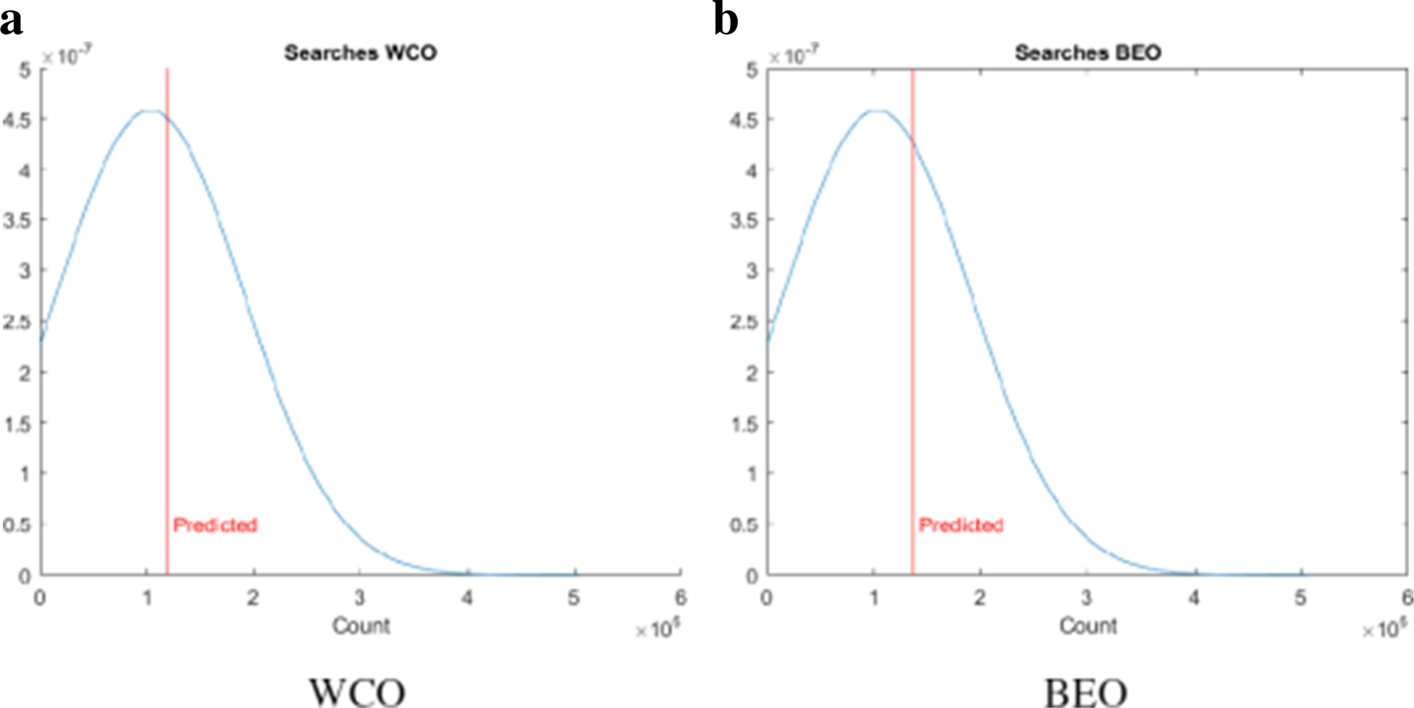
Estimated vs. actual searches for synthetic dataset

**Fig. 13:**
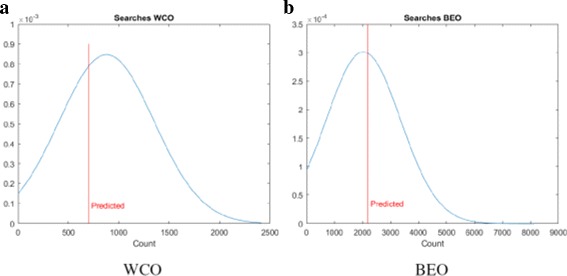
Estimated vs. actual searches for GDSC dataset

We then use the theoretical estimate outlined in “Lexicographic search analysis” section to predict the number of searches required by the LS algorithm. In all the scenarios, we are assuming *P*=5. The predicted value is plotted against the actual value fitted to a normal distribution and the plots are shown in Fig. 12 for synthetic data and Fig. 13 for the GDSC data. We note that the theoretical estimates are very close to the mean of the distributions confirming our savings estimate.

To explain the limited number of searches required for GDSC models as compared to synthetic models, note that $g^{wco}_{P}(l,\theta)$ and $g^{beo}_{P}(l,\theta)$ have gradual decreases with *l* for synthetic data as shown in Figs. 6 and 9 respectively whereas $g^{wco}_{P}(l,\theta)$ and $g^{beo}_{P}(l,\theta)$ for GDSC models have sharp decreases with *l* as shown in Figs. 7, 8, 10 and 11. Due to the sharp change in the probabilities, the expected number of searches required for LS for GDSC models (in the 600 to 2200 range) is significantly lower than the expected number of searches required for the synthetic models (in the 10^6^ range).

## Conclusions

In this paper, we have formulated the combination therapy design problem of maximizing efficacy while minimizing toxicity as an algorithmic search problem to find the optimal target set that maximally inhibit spatially heterogeneous cancer cell models while maintaining the effect along multiple normal cell models below a certain threshold. Our cell proliferation models are based on probabilistic target inhibition map (PTIM) framework [4–7] that consists of a series of blocks where each block contains a set of targets connected in parallel. To find the ideal target inhibition profile, we proposed a lexicographic search method to effectively search through all possible solutions. This method takes advantage of the properties of the PTIM to significantly reduce the number of potential solutions that we have to search through. We compare the performance and computational complexity of this method with other commonly used algorithms such as Genetic algorithms and Hill Climbing. We demonstrated the effectiveness of our algorithms using both synthetic models and models generated from Drug Sensitivity of Cancer Database. A theoretical analysis of the expected number of steps required by the Lexicographic Search process is included that was shown to provide a close approximation to actual observed search steps.

## Abbreviations

BEO: Best expected optimization
GA: Genetic algorithm
GACT: Genetic algorithm based combination therapy
GDSC: Genomics of drug sensitivity in cancer
HC: Hill climbing LS: Lexicographic search
PTIM: Probabilistic target inhibition map
WCO: Worst case optimization
